# Colour and melanopsin mediated responses in the murine retina

**DOI:** 10.3389/fncel.2023.1114634

**Published:** 2023-03-13

**Authors:** Joshua W. Mouland, Alex J. Watson, Franck P. Martial, Robert J. Lucas, Timothy M. Brown

**Affiliations:** ^1^Centre for Biological Timing, Faculty of Biology Medicine and Health, University of Manchester, Manchester, United Kingdom; ^2^Division of Diabetes, Endocrinology and Gastroenterology, Faculty of Biology Medicine and Health, University of Manchester, Manchester, United Kingdom; ^3^Division of Neuroscience and Experimental Psychology, Faculty of Biology Medicine and Health, University of Manchester, Manchester, United Kingdom

**Keywords:** colour opponency, melanopsin, RGCs, retinal ganglia cells, ipRGCs, intrinsically photoreceptive retinal ganglion cells, silent substitution, mouse

## Abstract

**Introduction:** Intrinsically photosensitive retinal ganglion cells (ipRGCs) integrate melanopsin and rod/cone-mediated inputs to signal to the brain. Whilst originally identified as a cell type specialised for encoding ambient illumination, several lines of evidence indicate a strong association between colour discrimination and ipRGC-driven responses. Thus, cone-mediated colour opponent responses have been widely found across ipRGC target regions in the mouse brain and influence a key ipRGC-dependent function, circadian photoentrainment. Although ipRGCs exhibiting spectrally opponent responses have also been identified, the prevalence of such properties have not been systematically evaluated across the mouse retina or yet been found in ipRGC subtypes known to influence the circadian system. Indeed, there is still uncertainty around the overall prevalence of cone-dependent colour opponency across the mouse retina, given the strong retinal gradient in S and M-cone opsin (co)-expression and overlapping spectral sensitivities of most mouse opsins.

**Methods:** To address this, we use photoreceptor isolating stimuli in multielectrode recordings from human red cone opsin knock-in mouse (Opn1mwR) retinas to systematically survey cone mediated responses and the occurrence of colour opponency across ganglion cell layer (GCL) neurons and identify ipRGCs based on spectral comparisons and/or the persistence of light responses under synaptic blockade.

**Results:** Despite detecting robust cone-mediated responses across the retina, we find cone opponency is rare, especially outside of the central retina (overall ~3% of GCL neurons). In keeping with previous suggestions we also see some evidence of rod-cone opponency (albeit even more rare under our experimental conditions), but find no evidence for any enrichment of cone (or rod) opponent responses among functionally identified ipRGCs.

**Conclusion:** In summary, these data suggest the widespread appearance of cone-opponency across the mouse early visual system and ipRGC-related responses may be an emergent feature of central visual processing mechanisms.

## Introduction

The search for the retinal mechanisms responsible for regulation of the mammalian circadian system lead to discovery of intrinsically photosensitive retinal ganglion cells (ipRGCs; Berson et al., 2002; Hattar et al., 2002), a cell type considered to be specialised for encoding ambient light intensity and driving subconscious and reflex, so-called “non-image-forming”, responses to light. More recently, it has emerged that this view likely represents a simplification of the properties and roles of ipRGCs. Hence, on one hand, new ipRGC subtypes have been discovered that project to parts of the brain mediating conventional aspects of vision (the dorsal lateral geniculate nucleus; dLGN) and roles for melanopsin in image forming vision have emerged (Brown et al., 2010, 2012; Ecker et al., 2010; Estevez et al., 2012; Schmidt et al., 2014; Allen et al., 2017, 2019; Stabio et al., 2018; Quattrochi et al., 2019). On the other hand, studies of the sensory properties of ipRGCs, central neurons receiving such signals and/or related non-image forming responses have suggested that variations in ambient light intensity are not the only type of visual information such cells may relay (Dacey et al., 2005; Chang et al., 2013; Walmsley et al., 2015; Mouland et al., 2017, 2021a; Hayter and Brown, 2018; Stabio et al., 2018). Hence, we recently showed that a substantial fraction of cells in the mouse suprachiasmatic nuclei exhibit opponent responses to selective stimulation of the two cone opsin classes and that the resulting colour-signals modulate the amplitude of circadian responses to light (Walmsley et al., 2015; Mouland et al., 2019). Further, we have found that substantial fractions of cells in both the pretectal olivary nucleus [PON, central relay regulating pupil responses (Hayter and Brown, 2018)] and visual thalamus that display evidence of ipRGC input similarly exhibit cone-opponent responses (Mouland et al., 2021a). Collectively then these data imply a strong association between ipRGC input and the presence of colour opponency, most parsimoniously explained by the existence of one or more subtypes of ipRGCs that themselves are colour opponent.

It has long been recognised that primate ipRGCs receive cone opponent signals (Dacey et al., 2005) and, more recently, evidence has emerged that two subtypes of mouse ipRGCs that could provide such colour opponent signals, M5 (Stabio et al., 2018) and M4/ON-α-RGCs (Chang et al., 2013; Schmidt et al., 2014). Of note however, while input from these ipRGC subtypes could potentially explain the co-occurrence of melanopsin and cone-opponent signals in the LGN and/or PON these subtypes are not known to provide significant projections to the SCN. Rather, input to the SCN is thought to primarily derive from M1-type ipRGCs (Baver et al., 2008). Previous studies have not found evidence of colour opponency in mouse M1 ipRGCs, raising questions about the potential origin of the colour-opponent responses that are commonly observed at the level of SCN neurons. It is also noteworthy that cone-opponent responses have been found in many pretectal and visual thalamic neurons that lack evidence of ipRGC input (Hayter and Brown, 2018; Mouland et al., 2021a), suggesting either that colour opponency is commonplace across RGCs in general, or present in a few classes that dominate input to such regions. To date, however, existing investigations of colour opponency at the level of the mouse retina have differed rather widely in their estimates of prevalence and proposed mechanisms (Ekesten et al., 2000; Ekesten and Gouras, 2005; Chang et al., 2013; Joesch and Meister, 2016; Stabio et al., 2018; Sonoda et al., 2020b; Szatko et al., 2020).

The capacity for colour discrimination in the mouse retina has traditionally been considered limited owing to the strong dorsal-ventral gradient in the (co)-expression of M- and S-cone opsins, whereby the ventral retina is dominated by S-opsin expression and the dorsal retina by M-opsin (Rohlich et al., 1994; Szel et al., 1994; Calderone and Jacobs, 1995; Applebury et al., 2000; Baden et al., 2013; Nadal-Nicolas et al., 2020). Accordingly, early studies comparing RGC responses to UV and green light, intended to preferentially stimulate S- and M-cone opsins (*λ*_max_ = 365 nm and 511 nm respectively) found only very few RGCs with spectral opponent responses (~2%; Ekesten and Gouras, 2005). Data from more recent studies suggest substantially higher fractions of UV-green opponent responses in RGCs, particularly those located in central and/or ventral parts of the retina (up to ~30% of all RGCs; Chang et al., 2013; Joesch and Meister, 2016; Szatko et al., 2020). One challenge in interpreting such data, however, is that the monochromatic comparisons used to date have not been fully selective for isolating responses from S- and M-cone opsins. Hence, on the one hand, all opsins exhibit moderate UV sensitivity due to their β-absorption band (Govardovskii et al., 2000; Nikonov et al., 2006), making it hard to ascribe an origin to any UV response. While, on the other, the strong spectral overlap between M-opsin and rhodopsin (*λ*_max_ = 498 nm) poses a challenge in ascribing a definitive origin to any “green” response (especially given recent data indicating that rods can continue to function under very high light levels; Tikidji-Hamburyan et al., 2015). Indeed, the surprisingly high prevalence of UV-green opponent responses identified in ventral parts of the retina (where M-cone opsin expression is scarce) have been interpreted as reflecting a non-canonical form of colour opponency involving comparisons between S-cones and rods (Joesch and Meister, 2016; Szatko et al., 2020). Such opponency has been observed at the level of bipolar cells and cones and proposed to originate through inhibition from rods *via* horizontal cells onto cones expressing S-opsin in the ventral retina (Szatko et al., 2020).

To address some of the challenges in interpreting data from more traditional approaches for assessing colour-opponency, one useful approach has been to employ a mouse line in which the native M-cone opsin is replaced by the human L-cone opsin (*Opn1mw^R^*), shifting cone spectral sensitivity far away from that of rods (λ_max_: 556 nm; Smallwood et al., 2003). In conjunction with multichromatic light sources, it, therefore, becomes possible to selectively manipulate the excitation of S- and/or L-cone opsin while nulling any contrast for other photopigments using the principles of “silent substitution” (Brown et al., 2012; Allen et al., 2014, 2017; Walmsley et al., 2015; Allen and Lucas, 2016; Dobb et al., 2017; Hayter and Brown, 2018; Mouland et al., 2019, 2021a,b). Using such techniques we have demonstrated widespread cone-mediated colour opponency across several major RGC target regions in the brain (Walmsley et al., 2015; Hayter and Brown, 2018; Mouland et al., 2021a). Here we now apply the same approaches to survey cone-based responses across the mouse retina *via* large-scale multi-electrode array recordings, to provide new insight into the prevalence of cone-mediated colour opponency at the level of retinal output and the extent to which this is relatively enriched among ipRGCs.

## Methods

### Mice

All experiments were in accordance with the UK Animals Scientific Procedures Act 1986 and European Directive 2010/63/EU. Eyes were collected from 20 *Opn1mw^R^* (RRID:MGI:2678771; 16 males and 4 females aged 2–10 and 2–4 months old respectively) and 3 *Opn1mw^R^* × *Opn4^−/−^* mice (2 female, 1 male aged 8–9 months). The *Opn1mw^R^* mice are from a C57Bl/6 background and have their native murine cone opsin replaced with the human L-cone opsin, which shifts the spectral sensitivity to longer wavelengths (λ_max_ 511 nm to 556 nm; Smallwood et al., 2003; Walmsley et al., 2015). Mice were housed under a standard 12:12 h light dark cycle without perturbation for at least 2 weeks prior to retinal collection. All animals had access to water and food *ad libitum*.

### Tissue collection

Mice were dark-adapted for 18 h prior to tissue collection. Mice we culled by Schedule 1 cervical dislocation in darkness with only a dim red head torch for illumination. Enucleated eyes were put into carboxygenated (95% O_2_/5% CO_2_) aCSF and dissected under a dissection microscope. The dissection took place in a dark room under illumination from the microscope at the dimmest setting (diffusing surface illuminated by incandescent bulb; ~11.5 log rod effective photons/cm^2^/s). Dissections consisted of piercing the edge of the cornea with a hyperdermic needle (25 gauge, Microlance, Becton Dickinson, Franklin Lakes, NJ). Then shearing along the ora serrata with vannas scissors (World Precision Instruments, Worcester, MA) and carefully removing the lens. The retina was then gently prised away from the eye cup with two pairs of Dumont #5 forceps (World Precision Instruments, Worcester, MA) The vitreous were carefully removed using the Dumount #5 forceps. The retina was incised multiple times at the edges in a Maltese cross motif to maximise planarization. Following dissection, retinas were immediately transferred to the recording apparatus (see below) where they dark adapted for 90 min prior to the start of data collection.

### Protocol

The retinas were mounted, RGC layer down, onto a transparent 256 channel multi-electrode array (256MEA200/30iR-ITO, Multichannel Systems GmbH, Reutlingen, Germany) and covered with a Cyclopore membrane (5-μm pores; Whatman Plc, Little Chalfont, UK) and a custom made anchor consisting of 2x stainless steel washers (~0.75 g) with parallel polyimide-coated fused silica capillaries (TSP320450; Polymicro Technologies, MOLEX LLC, Lisle, IL) attached to apply an even weight. To preserve physiological conditions carboxygenated artificial cerebro-spinal fluid (aCSF, NaCl: 118 mM; NaHCO_3_: 25 mM; Glucose: 10 mM; KCl: 3 mM; CaCl_2_: 2 mM; MgCl_2_: 1 mM; NaH_2_PO_4_: 1 mM; L-Glutamine: 0.5 mM) heated to 32°C in a water bath replenished the MEA chamber (with an initial flow rate of 4 ml/min) *via* a peristatic pump (PPS2, Multichannel Systems GmbH, Reutlingen, Germany). The temperature of the MEA was maintained at 32°C using a TC01 controller (Multi Channel Systems) regulating the temperature of a copper plate below the MEA. The retina was then left to dark adapt and settle for 90 mins prior to the protocol during which time the flow rate was increased slowly to between 4 and 6 ml/min.

### Pharmacology

The synaptic blockade was achieved using a combination of two glutamatergic antagonists: 80 μM 6,7-dinitroquinoxaline-2,3-dione (DNQX, Tocris Bioscience) and 130 μM (DL-AP4, Tocris Bioscience) in aCSF.

### Data acquisition and processing

Electrical activity was recorded *via* a USB-MEA256 amplifier (Multichannel Systems) using MC_Rack software (Multi Channel Systems) at 25 kHz sampling rate. The raw signal was filtered (2nd order Butterworth high-pass filter with 200 Hz cut off) and spikes passing threshold (6 standard deviations below the noise level) were timestamped and their waveforms (1 ms pre and post timestamp) captured. Single unit activity was isolated from recordings offline, using Offline Sorter V3.3.5 (Plexon, Texas, USA). Cross-channel artefacts were removed (events occurring simultaneously on >75% of channels) and single units were isolated manually by the presence of distinct clusters in principal component space and by reference to unit cross/autocorrelelograms, J3 and Davies-Bouldin sort-quality metrics (Supplementary Figure 2). The validity of unit separation isolation was confirmed by multivariate ANOVA and *post hoc* pairwise comparisons across units (*P* < 0.05; Offline Sorter).

### Light source

All full field light stimuli were produced using a custom- made light device. The light device consisted of three LEDs (λ_max_/Bandwidth 405 nm/12 nm, 530 nm/35 nm, 625 nm/17 nm. Thorlabs Inc. New Jersey), a high power blue LED (λ_max_ 460 nm Phlatlight PT-120 Series, Luminus Inc., Sunnyvale, California, USA) and a 3,000 k white LED (CBT-140-WHT, Luminus Inc., Sunnyvale, California, USA) with a yellow bandpass filter (FB580 Thorlabs Inc. New Jersey) which are driven through Labview (National Instruments, TX, USA) *via* an Arduino Due (Arduino, Ivrea, Italy). The light was delivered *via* a fibre optic, onto a dielectric mirror (CM1-E02, ThorLabs) and focused by a lens onto the back of the transparent MEA. Overall light intensity was varied over two orders of magnitude using a graded neutral density filter wheel (100FS04DV.4, Newport Corporation) controlled by an NSC200 controller system (Newport Corporation).

### Stimuli

**Figure 1:**
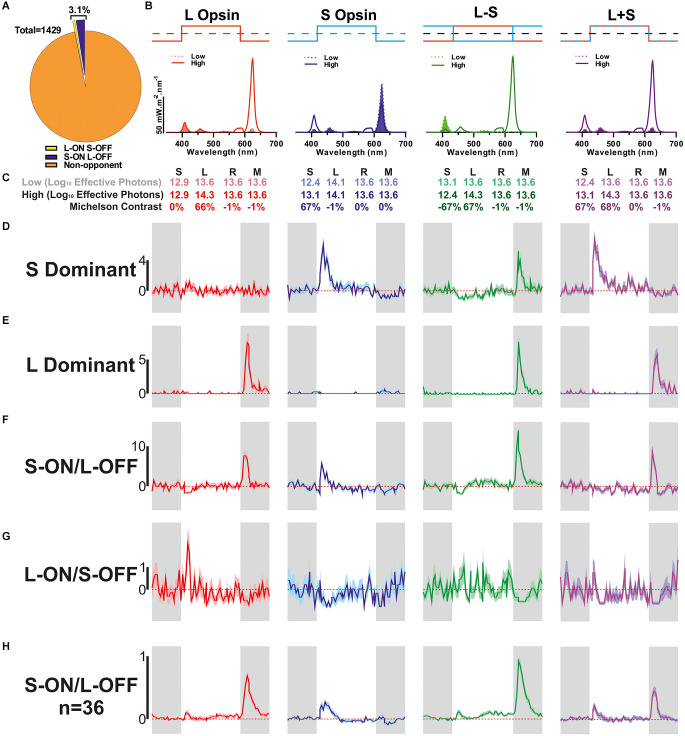
Detection of colour opponent units. **(A)** Categorisation of all units that were responsive to 67% Michelson contrast stimuli (*n* = 1,429) based on their response to L- and/or S-cone-selective contrast modulation (0.25 Hz square wave). **(B)** Spectral composition of the stimulus pairs used to isolate cone-based responses. Dashed filled line: low, solid line: high. **(C)** Relative photon absorbance (log_10_ photons) and Michelson contrast for each photopigment under each condition. **(D–G)** Mean ± SEM responses of example units for each of the cone isolating stimuli shown in **(B,C)** (*n* = 100 trials/stimulus); **(D)** non-opponent S-ON response, **(E)** non-opponent L-OFF response, **(F)** S-ON/L-OFF colour-opponent cell, **(G)** L-ON/S-OFF colour-opponent cell. **(H)** The mean ± SEM population response of S-ON/L-OFF (*n* = 36) colour opponent cells.

All light measurements were made at 1 nm intervals from 350 nm to 750 nm using a calibrated spectroradiometer (DMc150; Bentham Instruments Ltd, UK). Using the spectra absorption profiles of each murine photopigment and the human long wavelength sensitive photopigment, we calculated the relative photon absorption for each photopigment as the sum of photons at each wavelength weighted according to the relevant Govardovskii nomogram (Govardovskii et al., 2000; λ_max_: S opsin = 365 nm; L opsin = 556 nm; Rhodopsin = 498 nm; Melanopsin = 480 nm). This allowed us to produce metameric stimuli that were matched in “brightness” for chosen photopigments but differing for others. Stimuli designed to investigate colour were presented at 0.25 Hz as metameric pairs to produce photopigment specific steps (Figures 1B, 6A) and presented in interleaved blocks of 25 repeats (total 100 repeats per stimuli). Mel. High/Low stimuli (Figure 4A) were presented as 10 s steps from darkness (80 s ISI, 10 repeats, stimuli interleaved). Extrinsic signals to RGCs were detected using either the UV LED alone (λ_max_ 405 nm, 0.5 Hz; relative photon absorbance in log_10_ photons; S Opsin: 14.4, L Opsin: 14.4, Rod: 14.5, Mel:14.6) or the UV LED in conjunction with the red LED (λ_max_ 625 nm, 0.5 Hz; combined relative photon absorbance in log_10_ photons; S Opsin: 14.4, L Opsin: 14.7, Rod: 14.5, Mel:14.6). Activation of ipRGCs under synaptic block was made using narrowband 460 nm light (10 s, ISI: 90 s, 10 repeats, relative photon absorbance in log_10_ photons; S Opsin: 12.6, L Opsin: 15.1, Rod: 15.6, Mel: 15.7).

**Figure 2:**
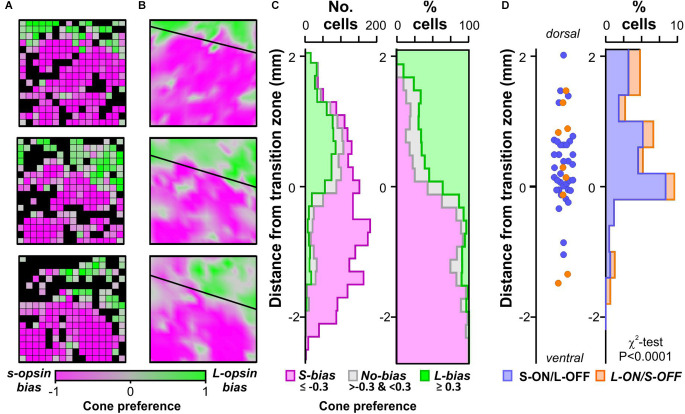
Distribution of colour opponency across the retina. **(A)** Population level cone preference from three 256-channel MEA recordings (spanning 3 × 3 mm of retina). Black colour indicates electrodes where we did not detect significant modulations in multiunit firing to either L- or S-opsin isolating stimuli. **(B)** Interpolated map of retina cone-preference from experiments in **(A)** showing opsin transition zone modelled by linear fitting. **(C)** Spatial distribution of isolated units with strong S-opsin bias, no bias, or strong L-opsin bias relative to modelled opsin transition zone (*n* = 1,429 cells from 25 retinas). Left panel shows numbers of isolated cells, right panel shows same data as a proportion of total for that location. **(D)** Spatial distribution of isolated colour opponent units relative to modelled opsin transition zone (*n* = 36 S-ON/L-OFF and *n* = 9 L-ON/S-OFF cells). Left panel shows estimated locations of individual cells, right panel shows binned proportions of colour opponent cells relative to all cells isolated within that region (data analysed by χ^2^-test).

**Figure 3:**
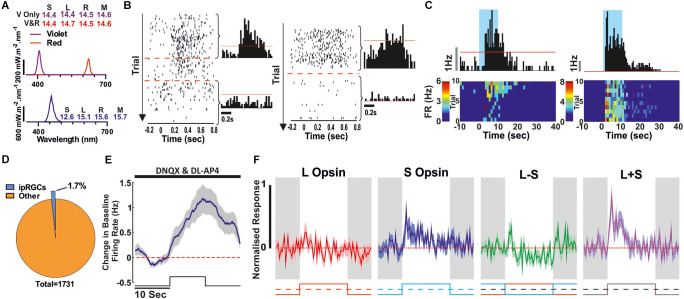
Synaptic blockade (DNQX and DL-AP4) to confirm ipRGCs. **(A)** Spectra and Relative photon absorbance (log_10_ photons) for each photopigment for the stimuli used here to check for extrinsic responses (rod or cone driven, top) and intrinsic responses (bottom). **(B,C)** Two example ipRGCs. **(B)** Rod/Cone mediated responses to a 500 ms light step (ISI: 500 ms; Violet or Violet and Red light) before and during the drug application which abolishes the RGC responses from the outer photoreceptive layer. *Left* raster plot, red lines denote the first and last 200 trials that were used to produce the before and after histograms to the *right*. The red line on the histograms denotes the 99% confidence interval that the response is above baseline firing rate. **(C)** Corresponding response to a 10 s light step (Blue light; 90 s ISI) in the presence of the synaptic blockade. *Above* Perievent histogram, red line denotes the 99% confidence interval that the response is above baseline firing rate. Below is the corresponding heatmap showing the trial by trial response. **(D–F)** Combined data from all ipRGCs classified this way. **(D)** Proportion of all light responsive units that showed a light response following synaptic blockade. **(E)** Mean ± SEM population response to a 10 s light pulse whilst under synaptic blockade (*n* = 30, 2.5 s smoothing). **(F)** The Mean ± SEM cone responses from this ipRGC population in the absence of synaptic blockade (using stimuli illustrated in Figures 1B,C).

**Figure 4:**
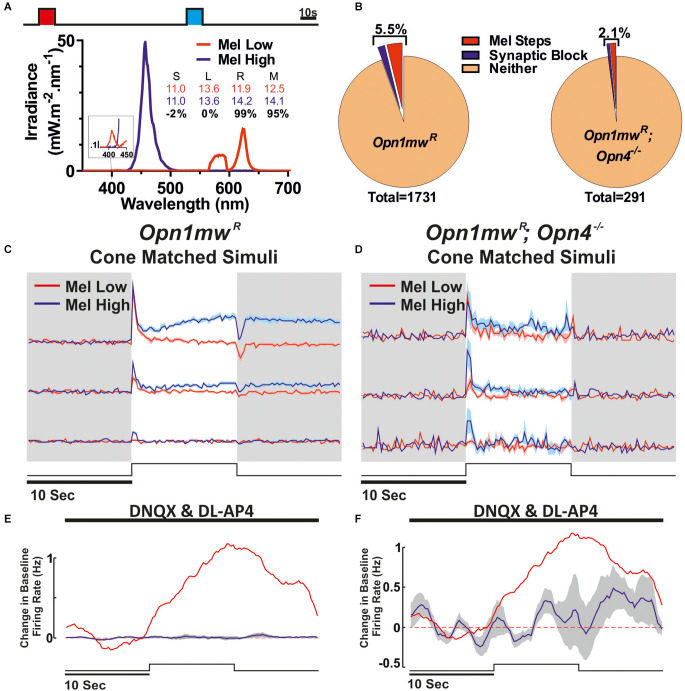
Identification of melanopsin responses *via* cone-isoluminant light steps. **(A)** Stimuli presented to the mice. Melanopsin High (blue) and Low (red) stimuli were presented as 10 s light steps from darkness (80 s ISI). Respective spectra and relative photon absorbance (log_10_ photons) for each photopigment is shown below. Michelson contrast between stimuli shown in black. **(B)** Proportions of all cells passing our criteria for ipRGC classification based on response under synaptic blockade or comparison between Mel High and Low steps in *Opn1mw^R^* (*left*) and *Opn1mw^R^; Opn4^−/−^* (*right*) mice. Data were collected in *Opn1mw^R^* mice **(C,E)** and animals which lacked functional melanopsin expression (*Opn1mw^R^; Opn4^−/−^*; **D,F**). **(C,D)** Mean ± SEM response of units that were deemed melanopsin positive from the Mel high vs. low steps from darkness. (**C**: *n* = 66/1,735, **D**: *n* = 4/291). **(E,F)** Mean ± SEM response under synaptic blockade for putative ipRGCs to a 10 s light pulse (2.5 s smoothing; stimuli and spectra Figure 3A *bottom*). **(E)** Mean ± SEM response of cells shown in **(C)**. Red trace is the mean response of ipRGCs identified under synaptic block Figure 3E. **(F)** All cells passing either of our ipRGC classification criteria in red cone MKO mice (*n* = 6).

**Figure 5:**
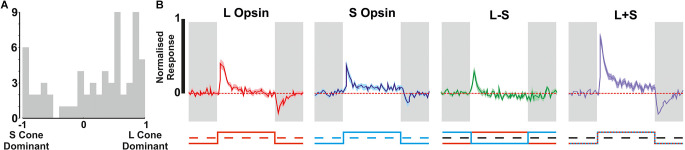
Cone mediated responses in the second subpopulation of ipRGCs. **(A)** Distribution of S- vs. L- cone preference across putative non-M1 ipRGCs isolated using Mel High vs. Low steps. **(B)** Mean ± SEM cones responses of the putative non-M1 ipRGC population to various cone isolating stimuli (*n* = 66).

**Figure 6:**
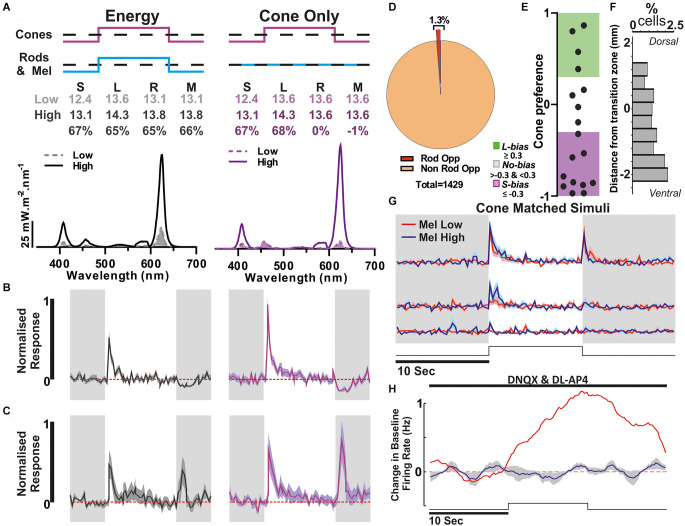
Putative Rod opponency. **(A)** Paired light stimuli used to detect Rod opponency. *Above*: Relative photon absorbance (log_10_ photons) and Michelson contrast for each photopigment. *below* spectra, dashed filled line: low, solid line: high. **(B)** An example unit showing evidence of rod-opponency, where responses to L+S contrast were greater than the response to equivalent contrast applied to both rods and cones. **(C)** Mean ± SEM of all cells with evidence of rod opponency (*n* = 18/1,429). **(D)** Proportion of units that responded to the 67% Michelson contrast steps that were classified as rod opponent. **(E)** Cone preference of putative Rod Opponent cells (*n* = 18). **(F)** Percentage of cells that are classed as rod opponent at each location. **(G,H)** Population data from all units displaying rod opponency. **(G)** Response to Mel high and Mel low steps from darkness (Mel high: blue, Mel low: red). **(H)** Response to a 10 s light step (Monochromatic Blue light, 90 s ISI) in the presence of synaptic blockade (spectra and irradiance as used in Figure 3).

### Analysis

All analysis was performed using custom Matlab scripts and plotted using either Matlab (R2019a: Mathworks, MA, USA) or GraphPad Prism (8.1.2, GraphPad Software Inc., CA, USA).

Units were deemed light responsive if they showed a robust change (One sample *t*-test: *p* < 0.0025) in firing from baseline to any single stimuli or significant responses (One sample *t*-test: *p* < 0.05) over multiple (≥3) stimuli. For the 0.25 Hz contrast stimuli and the 10 s steps from darkness the 500 ms immediately after light onset and offset was compared with baseline firing. Of these light responsive units only those that were light responsive to the 67% Michelson contrast steps (*n* = 1,429) were further analysed for colour opponency.

Units were classified as colour opponent under two conditions. The first condition was that the cell had a robust response (One sample *t*-test: *p* < 0.0025) to both the S-opsin and the L-opsin only stimuli but with different polarity, as determined by the timing of the peak in firing rate. The second condition was that the cell had a robust response to the L−S stimuli (One sample *t*-test: *p* < 0.0025) that was significantly greater than the response to the L+S stimulus (T-test: *p* < 0.0025), and the equivalent S/L-opsin response alone. Cells were considered rod opponent if there was a robust response to the L+S+ stimuli (One sample *t*-test: *p* < 0.0025) and the L+S+ response was greater than the Energy response (T-test, *p* < 0.0025). Cone opsin preference was determined using the following formula (L_RA_ − S_RA_)/(L_RA_+S_RA_) where L_RA_ and S_RA_ are the response amplitudes to the L opsin only stimuli and S opsin only stimuli respectively.

For identifying melanopsin responses in the presence of synaptic blockade we used a 10 s sliding window from the start of light on to 15 s after light off. Cells were considered responsive when peak firing in this window was significantly greater than the 10 s preceding light on (*t*-test: *p* < 0.0025). For classification of additional putative ipRGCs, cells were deemed responsive if they displayed an increased sustained (*t*-test: *p* < 0.0025) or prolonged (*t*-test: *p* < 0.0025) response to light under the Mel high vs. Mel low condition but lacked an equivalent increase in the initial (rod/cone-dominated) transient response.

## Results

### Cone-based responses and retinal colour opponency

We recorded extracellular activity from the ganglion cell layer (GCL) of 25 isolated retinal preparations from red cone mice whilst presenting a range of light stimuli to assess different features of photoreceptive input of the recorded neurons. Across these preparations, we isolated *n* = 1,731 neurons that responded to one or more test stimuli (see Methods). In our first stimulus paradigm, we aimed to elucidate the impact of cone-mediated inputs and the occurrence of colour opponency, by presenting a range of stimuli (Figure 1B) designed to provide substantial contrast (67%) for S- and/or L-cone opsin but negligible contrast (<1%) for other “silenced” photoreceptors (Figure 1C). The majority of GCL neurons detected in these experiments (*n* = 1,429/1,731; ~83%) exhibited a significant response to one or more of these 67% contrast stimuli. These responsive units were further classified according to the nature of the changes in firing rate evoked by our cone-isolating stimuli (Figures 1A,D–H, See Section “Methods”). The majority of such cells (*n* = 1,384/1,429; ~96.9%) were classified as non-opponent and either only responded to contrast targeting one of the two cone-opsin classes (see Figures 1D,E for examples of units with S-ON unit or L-OFF responses) or displayed responses of the same polarity (i.e., ON or OFF) to both L- and S-opsin isolating stimuli. By contrast, we did identify a small subset of units that displayed evidence of colour opponent responses (*n* = 45/1,429; ~3.1%; Figure 1A). Such cells either displayed changes in firing rate of opposite polarity to L- and S-opsin isolating stimuli (see Figures 1F,G for examples of S-ON/L-OFF and L-ON/S-OFF opponency) or lacked detectable responses to one of these stimuli but displayed significantly greater modulations in firing when presented with chromatic, L−S, modulations vs. achromatic L+S modulations in cone excitation. The majority of colour opponent units identified in these experiments exhibited S-ON/L-OFF responses (Figure 1H; *n* = 36). By contrast, cells that met the objective criteria for classification as L-ON/S-OFF were observed more rarely and tended to exhibit only weak opponency (Figure 1G, Supplementary Figure 1A, *n* = 9). Indeed, a majority (*n* = 6/8) of such cells lacked a readily detectable response to one of the two single cone opsin isolating stimuli and were instead classified as opponent based on a significant reduction in response to cone luminance (L+S) vs. chromatic (L−S) modulation (and an L-ON or S-OFF bias to their responses; Supplementary Figure 1B).

We next investigated the retinal location of these cone-driven colour opponent cells, specifically by reference to the gradient in S- vs. M/L-opsin expression, which various studies have reported to impact the appearance of colour opponency (Chang et al., 2013; Joesch and Meister, 2016; Szatko et al., 2020). Here we functionally estimated the positions of recorded cells relative to the transition zone between S- and M/L-dominated, ventral and dorsal retina, based on multiunit firing responses to cone opsin-isolating stimuli detected across our 256 channel electrode arrays (which spanned 9 mm^2^ of the retina). Specifically, we derived a cone-preference index based on relative response to S- vs. L-opsin isolating stimuli at each electrode (Figure 2A), and modelled the transition zone based on a linear fit through the points at which population responses to the two stimuli were equally matched (Figure 2B). We then calculated the dorsal-ventral distance of each isolated single unit from this transition zone. As expected then, L-opsin biased units (cone-preference ≥0.3) were strongly clustered dorsal to the transition zone, whilst more ventral locations became progressively enriched for highly S-opsin biased units (cone preference ≤ −0.3; Figure 2C). Nonetheless, it was possible to find occasional S-opsin-biased cells dorsally and, to a lesser extent, L-opsin biased cells ventrally. Significantly, this analysis also revealed that the locations of colour opponent units were widely distributed across dorsal and ventral axis of the retina (Figure 2D). Of note, however, there was a non-uniform distribution in the relative proportion of cells exhibiting cone-opponent responses (Figure 2D, χ^2^-test, *P* < 0.001), with the greatest concentration of cells found around the opsin transition zone (where both cone opsin classes are abundant) and very low proportions in ventral parts of the retina, where M/L-opsin expression is scarce (Baden et al., 2013; Nadal-Nicolas et al., 2020; Mouland et al., 2021a).

### Identification of ipRGCs

Having extensively surveyed cone-based responses and the occurrence of colour opponency across the retina of *Opn1mw^R^* mice, we next set out to identify ipRGCs. We started by drawing on the “gold standard” method of looking for cells that retained light-driven changes in firing in the presence of synaptic blockers (DNQX and DL-AP4) to abolish rod/cone-mediated responses (Berson et al., 2002; Hattar et al., 2002; Lucas et al., 2003; Schmidt et al., 2008). Shortly after applying the synaptic blockers all responses to brief (500 ms) bright light steps designed to robustly activate rod and cone inputs (Figure 3A) were abolished (Figure 3B). However, when presented with longer (10 s) light steps of bright 460 nm light (log_10_ effective photons; S Opsin: 12.6, L Opsin: 15.1, Rod: 15.6, Mel: 15.7), the hallmark sluggish increases in firing that characterise melanopsin-driven light responses (mean ± SEM time to peak: 8.7 ± 1.1 s) were observed in a small subset of cells (*n* = 30/1,731; ~1.7%; Figures 3B–E). This proportion aligns closely with original estimates of the proportion of melanopsin-expressing RGCs based on approaches that preferentially label the M1 subtype (Hattar et al., 2002) but is substantially less than the proportions revealed using later reporter constructs to identify additional subtypes with weaker melanopsin expression (Brown et al., 2010; Ecker et al., 2010). It is possible, therefore, that the approach used here preferentially reveals the M1-subtype of ipRGC. Moreover, in keeping with previous studies that have investigated outer retinal inputs to M1 cells in mouse retina (Schmidt et al., 2008; Weng et al., 2013), we found that the cone response of these ipRGCs (tested prior to the synaptic blockade) was relatively weak (Figure 3F) with no evidence of colour opponency across any of the identified units.

Given that MEA recordings of light responses under the condition of synaptic block appear insufficient to reveal the full complement of ipRGCs [presumably due to lower levels of melanopsin expression in non-M1 cells; review (Aranda and Schmidt, 2021)], we also employed an alternate, potentially more sensitive classification method. For this, we adapted approaches we have previously used to identify melanopsin responses in brain regions targeted by ipRGCs (Hayter and Brown, 2018; Mouland et al., 2021a), by designing stimuli that were matched in brightness for cones but differed in their brightness for melanopsin and rods (Figure 4A). These were then presented as interleaved 10 s steps from darkness, across an intensity range where even the “Mel low” stimulus was expected to drive transient rod saturation (Figure 4A, 80 s ISI). Given differences in the temporal kinetics of inner and outer retinal photoreception, initial transient increases in firing evoked by such light steps should be dominated by rod/cone inputs while any melanopsin component (if present) should emerge after extended light exposure. Accordingly, we selected cells where tonic components of the response and/or continued post-stimulus firing were preferentially enhanced for the Mel High stimulus (see Methods for full details). Using this approach, we detected a further 66 cells (~3.8% of the 1,731 light responsive neurons surveyed) that displayed evidence of melanopsin-driven responses (Figure 4C), i.e., putative ipRGCs, across the cells that lacked robust responses to light under synaptic blockade (Figure 4E). Together, then our two methods suggest ~5.5% (*n* = 96/1,731) of the cells we sampled were ipRGCs (putatively comprising an ~2:1 ratio of non-M1 to M1 subtypes), a total proportion very similar to recent estimates of ipRGC numbers using reporters that label all subtypes (Brown et al., 2010; Ecker et al., 2010; Sand et al., 2012; Figure 4B). Interestingly, in most cases (*n* = 28/30) the population of cells identifiable under synaptic block did not meet our ipRGC classification criteria in this alternate paradigm. Rather, subpopulations of those cells either had constitutively high firing, became largely silent, or exhibited only a brief increase in spiking at stimulus onset. We suspect these observations reflect a combination of factors that are especially pronounced in the M1 ipRGCs (a comparatively high sensitivity, their persistent response and propensity to go into depolarisation block; Zhao et al., 2014; Milner and Do, 2017; Lee et al., 2019) and which impairs our ability to reliably identify differences in their response to melanopsin high vs. low stimuli under this second approach.

To validate our two approaches for identifying ipRGCs, we also performed equivalent experiments in retinas from melanopsin knockout red cone mice (*Opn1mw^R^*; *Opn4^−/−^*, *n* = 4 retinas from three mice). Here, we did find a small number of units that nominally passed our classification criteria based on the presence of increased firing following a light pulse under synaptic blockade (*n* = 2/291) or difference in response to Mel High vs. Low steps (*n* = 4/291). Importantly, however, the proportions revealed were significantly reduced (χ^2^-test, *P* < 0.05), and the identified “responses” far weaker compared to red cone mice with functional melanopsin (Figures 4B,D,F). Nonetheless, these data raise the possibility that our approaches for identifying putative ipRGCs in red cone mice may somewhat overestimate the “true” proportion displaying functional evidence of melanopsin input. On balance, for subsequent analysis of cone inputs, we considered it preferable to use this existing classification scheme, rather than a more stringent set of criteria that fully excluded false positives at the risk of also losing true positives.

We, therefore, went on to examine the cone-based responses of the additional putative non-M1 ipRGCs identified in red cone mice using the Mel High vs. Low stimulus comparison. As expected this population of cells exhibited strong ON-type responses to cone-isolating stimuli (more robust than those of ipRGCs identified based on light responses under synaptic block), including cells with strong S- or L-cone opsin bias and cells with more balanced input from both opsin classes (Figures 5A,B). Importantly, across this population, we observed minimal evidence of colour opponency. A small subset of these putative non-M1 ipRGCs passed our criteria for opponency (L-ON/S-OFF *n* = 2/66, S-ON/L-OFF *n* = 1/66) including the example cell shown in Figure 1G. Overall then, only ~3% of putative ipRGCs identified (*n* = 3/96) displayed any evidence of colour opponency, matching the overall proportion of RGCs we identify with this property (Figure 1A).

While our estimates of the prevalence of RGC colour opponency correspond well with original suggestions (Ekesten and Gouras, 2005), they are substantially lower than recent estimates of RGCs exhibiting opponent calcium responses to short- vs. medium-wavelength light (Szatko et al., 2020). Given suggestions that rods may play important roles in driving opponent responses in mouse RGCs (Joesch and Meister, 2016; Szatko et al., 2020), we further assessed whether there was any evidence for widespread rod-cone opponency among the RGCs recorded here. To this end, we compared the responses of GCL neurons to our L+S stimulus with those evoked by a spectrally neutral modulation providing equivalent contrast for L- and S-cone opsin but also providing a high (67%) contrast for rods (Figure 6A). We found very few units (*n* = 18/1,429 contrast responding cells; ~1.3%) that showed a significantly reduced response to this latter “energy” stimulus compared to the cone-selective L+S stimulus (Figures 6B–D). As one might expect for meaningful rod opponency, the cone mediated responses of such cells are largely S-opsin driven (Figure 6E; *n* = 10/18 < −0.3) allowing the potential for S-opsin vs. Rod opponency. Moreover, the majority of these units were located in the ventral retina (Figure 6F), consistent with findings from recent studies implicating rods in observed spectral opponent mechanisms. We should note here, that there is also a difference in melanopsin contrast between the L+S and energy stimuli used above. This is most unlikely to account for any difference in response observed, however, both due to the comparatively high temporal frequency employed and because none of the cells classed as rod-opponent exhibited evidence of melanopsin-driven responses in other paradigms (Figures 6G,H).

## Discussion

Using large-scale multielectrode recording approaches, we here show that the prevalence of cone-driven colour opponency among mouse GCL neurons (~3%) is remarkably low. In interpreting these data it is important to consider the fact that a relatively high proportion of cells within the ganglion cell layer (~50%) are estimated to be displaced amacrine cells (dACs; Schlamp et al., 2013), although many of these are non-spiking. While the exact proportions are unclear, this includes the most numerous dAC subtype (Starburst amacrine ~66% of dACs; Zhou and Fain, 1996; Müller et al., 2007) and at least one other subtype (A17 amacrine cell; ~3% of dACs; Menger and Wassle, 2000; Müller et al., 2007). We estimate therefore that the majority of GCL neurons we record (>85%) are RGCs. Given, also, previous data suggesting colour-opponency is similarly common among RGCs and dACs (Szatko et al., 2020), we consider our data a reliable estimate of the occurrence of colour-opponenecy among RGCs.

Consistent with one of the major proposed mechanisms of cone-opponency in the mouse retina, we find cone-opponent cells are specifically enriched around the dorsal-ventral cone opsin transition zone (Chang et al., 2013). Also in line with previous suggestions (Joesch and Meister, 2016; Szatko et al., 2020), we further find some evidence of rod-cone opponency, especially in more ventral retinal locations, although this property is even more rare under our experimental conditions (~1% of RGCs). Collectively, then, these estimates of the extent of retinal colour opponency are at the lower end of those provided by studies that have used approaches which do not unambiguously distinguish between rod and cone-based responses (Ekesten and Gouras, 2005; Szatko et al., 2020). Most significantly, however, they are markedly lower than the proportions of neurons (~30%) displaying cone-mediated opponency in recordings from major RGC target neurons using near-identical approaches to those employed here (Walmsley et al., 2015; Hayter and Brown, 2018; Mouland et al., 2021a). Furthermore, whilst we have routinely observed a co-occurrence of strong melanopsin-driven responses and the existence of opponency in such central recordings (Walmsley et al., 2015; Hayter and Brown, 2018; Mouland et al., 2021a) we here find no evidence that cone (or rod) opponency is enriched among ipRGCs, nor evidence of cells that have both strong melanopsin responses with robust strong colour opponency. Collectively this aligns with previous suggestions (Mouland et al., 2021a) that central mechanisms may play a significant role in generating colour opponency and/or the integration of colour and melanopsin signals within the brain.

One striking feature of the present findings is the much lower proportion of colour-opponent neurons we identified, especially in ventral retinal locations, compared to another recent large-scale survey of UV-green spectral opponency using calcium imaging (Szatko et al., 2020). The strong enrichment of UV-green spectral opponency in the M-opsin sparse ventral retina observed in that latter study was ascribed to potential rod vs. S-opsin opponency. While we here find data in support of such a mechanism, the fraction of GCL neurons exhibiting this property is very low compared to the ~30% reported previously (Szatko et al., 2020). This could, in principle, reflect a greater selective for RGCs vs. dACs in the present study (since calcium imaging can also detect non-spiking cells), although, as alluded to above, data from Szatko et al suggests colour opponency is also common in putative RGCs identified by functional properties. Accordingly, the most likely reason for the apparent discrepancy relates to differences in experimental design, with our stimuli primarily intended to isolate cone-mediated colour opponency and hence around 10-fold brighter than the stimuli in that previous study (Szatko et al., 2020). Indeed, an earlier study that reported rod-cone opponency in a specific RGC type (Joesch and Meister, 2016), found this was present at background light intensities similar to those employed by Szatko and colleagues but largely absent at light intensities more similar to those employed here (~10^5^ R*/s). Of course, it also remains possible that the imaging approach employed by Szatko et al. (2020) is more amenable to detecting colour-opponent influences on RGC physiology than the approaches employed here. Nonetheless, the present data certainly indicate that, at the level of RGC spike output, spectral opponency is relatively rare under conditions equivalent to those that produce robust cone-mediated opponent responses in the brain and behaviour.

Behavioural assessments of colour discrimination in mice suggest that sensitivity is non-uniform across the visual field (Denman et al., 2018). Specifically, the capacity of mice to discriminate UV vs. green was maximal at ~50° above the horizon. After correcting for the tilt of the mouse eye (~22°upwards) this corresponds fairly closely to the transition zone of retinal M- vs. S-cone expression (Sterratt et al., 2013) and where we find the highest density of colour opponent neurons. Indeed, while we do find some colour opponent neurons in dorsal retinal locations equivalent to those where Denman et al. (2018) cease to be able to detect colour discrimination, the present findings are broadly consistent with spatial variation in chromatic sensitivity seen at the behavioural level.

Another notable aspect of our data is the relative lack of cells that display evidence of melanopsin-driven responses and also exhibit colour-opponency. We utilised two methods for identifying putative ipRGCs in our recordings. The first approach, using synaptic blockade to directly identify RGCs with intrinsic photosensitivity has been used widely (Berson et al., 2002; Hattar et al., 2002; Lucas et al., 2003; Schmidt et al., 2008). In our extracellular recordings, only ~1.7% of cells are identifiable as ipRGCs using this method, which corresponds to the proportion identified using methods that preferentially identify the M1-subtype but is substantially lower than the total (~5% of RGCs) identified with more sensitive reporters (Hattar et al., 2006; Brown et al., 2010; Ecker et al., 2010; Sand et al., 2012). We hypothesise, therefore, that the synaptic block approach principally reveals M1 ipRGCs in our MEA recordings, which have the highest density of melanopsin expression. We suspect this is unlikely to constitute a “pure” population of M1 cells, however, since the response latencies revealed here, while faster than those reported for M3-M5 cells, are on average slower than expected for only M1 cells identified in patch recordings (Tu et al., 2005; Zhao et al., 2014). Nonetheless, consistent with the view this population is enriched for M1 cells, we found their response to cone-modulating stimuli was consistently weak. Moreover, in line with a previous study specifically targeting the M1 subtype (Weng et al., 2013), we found no evidence that such cells exhibited cone-mediated colour opponent responses. Hence the widespread appearance of cone opponent responses in SCN neurons (Walmsley et al., 2015) is most unlikely to be directly inherited from the M1 ipRGC subtype that dominates retinal input to that structure (Beier et al., 2021).

To overcome limitations of the synaptic block approach and identify other ipRGC subtypes with lower melanopsin expression (as well as stronger rods/cone mediated responses), we used a second approach of comparing responses to cone-isoluminant and (transiently) rod-saturating light steps that differed in melanopic irradiance. We have used this approach to effectively identify melanopsin-driven responses in the PON and visual thalamus (Hayter and Brown, 2018; Mouland et al., 2021a) and here this allowed us to identify a further subset of putative ipRGCs (~3.8% of cells) such that the total proportion of identified here (~5.5%) aligns with that expected using sensitive neuroanatomical reporters (Brown et al., 2010; Ecker et al., 2010). We suspect this latter population of cells is not readily identifiable under synaptic blockade because of their smaller intrinsic light responses (Zhao et al., 2014) are insufficient to substantially change firing in their own right (at least for the stimuli applied here) but is enough to modulate synaptically driven inputs. It is also noteworthy here that different ipRGC subtypes appear to employ different downstream signal transduction pathways (Jiang et al., 2018; Chen et al., 2023) that could, in principle, favour modulation of incoming synaptic signals vs. intrinsically driving spiking responses. In either case, among this group of putative non-M1 ipRGCs we do find evidence of a few cells that display evidence of weak cone-opponency, but certainly no more frequently than we find such responses across the total RGC population. This is somewhat surprising given previous reports that M5 ipRGCs display S-ON opponent responses and data suggesting that some M4/ON-α-RGCs might display S- or M-ON opponent responses depending on retinal location (Chang et al., 2013; Stabio et al., 2018; Sonoda et al., 2020b).

The relative lack of colour opponency among putative ipRGCs identified here, especially the S-ON opponent responses previously reported for M5 cells, suggests that our methods for identifying ipRGCs may fail for some with low melanopsin expression. Regardless, it is striking that using essentially identical approaches to those employed here we reliably identify many neurons in the brain that exhibited both colour opponency and robust melanopsin-driven responses (Walmsley et al., 2015; Hayter and Brown, 2018; Mouland et al., 2021a). While it remains possible that a very scarce ipRGC type is highly connected and influential at the central level, the absence of neurons with such properties in the present study strongly suggests one or more forms of convergent input must be important for the sensory properties we observe in our central recordings. This could take the form of separate RGCs providing cone-opponent and melanopsin-dependent features of the response, separate ON and OFF (ip)RGCs providing inputs differentially biased to S- or M/L-cone opsins, or more complex network mechanisms. Indeed, in the case of the SCN, the recent finding that a subset of ipRGCs uses GABA as a neurotransmitter (Sonoda et al., 2020a) raises the intriguing possibility that convergent input from excitatory and inhibitory ipRGCs with differing cone preference might contribute to the cone opponency observed there. In any case, existing evidence is certainly consistent with the possibility that subsets of both SCN and visual thalamic cells can receive convergent input from more than RGC type (Howarth et al., 2014; Walmsley and Brown, 2015; Rompani et al., 2017; Liang et al., 2018), proving a substrate that could support the types of mechanisms suggested above.

In conclusion, our findings add new insight into colour processing in the mouse visual system and the contributions of ipRGCs. In addition to supporting previous reports of the existence of rod-cone opponency in the mouse retina (albeit very rare under our experimental conditions), we show that cone-driven colour opponency is far less common across RGCs, including ipRGCs, than it is in major target regions for such cells. Our data, therefore, add weight to previous suggestions that central mechanism may play an especially important role in colour processing across both conventional and non-image-forming aspects of mouse visual function (Walmsley et al., 2015; Mouland et al., 2021a).

## Data availability statement

The raw data supporting the conclusions of this article will be made available by the authors, without undue reservation.

## Ethics statement

The animal study was reviewed and approved by UK Animals Scientific Proceedures Act 1986 and institutional animal ethics committee.

## Author contributions

TB, RL, and JM designed the experiments and wrote the manuscript. FM and JM constructed and calibrated the experimental apparatus. JM and AW performed the experiments. JM and TB performed the analysis. All authors contributed to the article and approved the submitted version.
