# Supplementary material for: Colour and melanopsin mediated responses in the murine retina
Source: Front Cell Neurosci. 2023 Mar 13;17:1114634. doi: 10.3389/fncel.2023.1114634 (PMC10040579; doi:10.3389/fncel.2023.1114634)
Supplement: Supplementary file 1 [file Data_Sheet_1.docx]

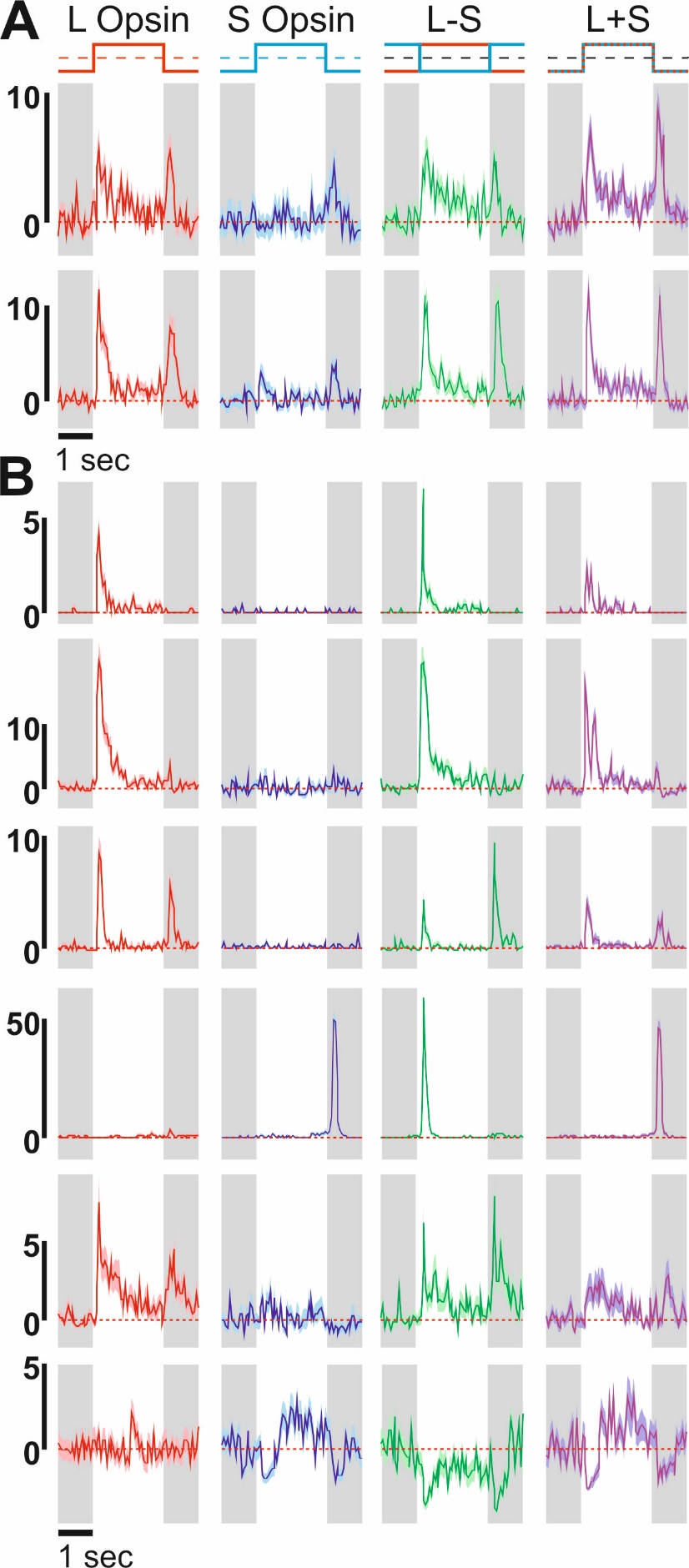


**Supplemental Figure 1. Variability in L-ON/S-OFF colour opponent units.** All units classified as L-ON/S-OFF units using the stimuli in Fig1B. **A** Units that showed an ON biased response to L-opsin and OFF biased response S-opsin selective stimuli (p<0.0025, n=3/9, additional unit showed in Fig1G.) **B** Units that lacked a response to one of the cone opsin isolating stimuli but exhibited a significantly larger amplitude response to Chromatic (L-S) vs achromatic (L+S) modulation (p<0.0025, n=6/9).


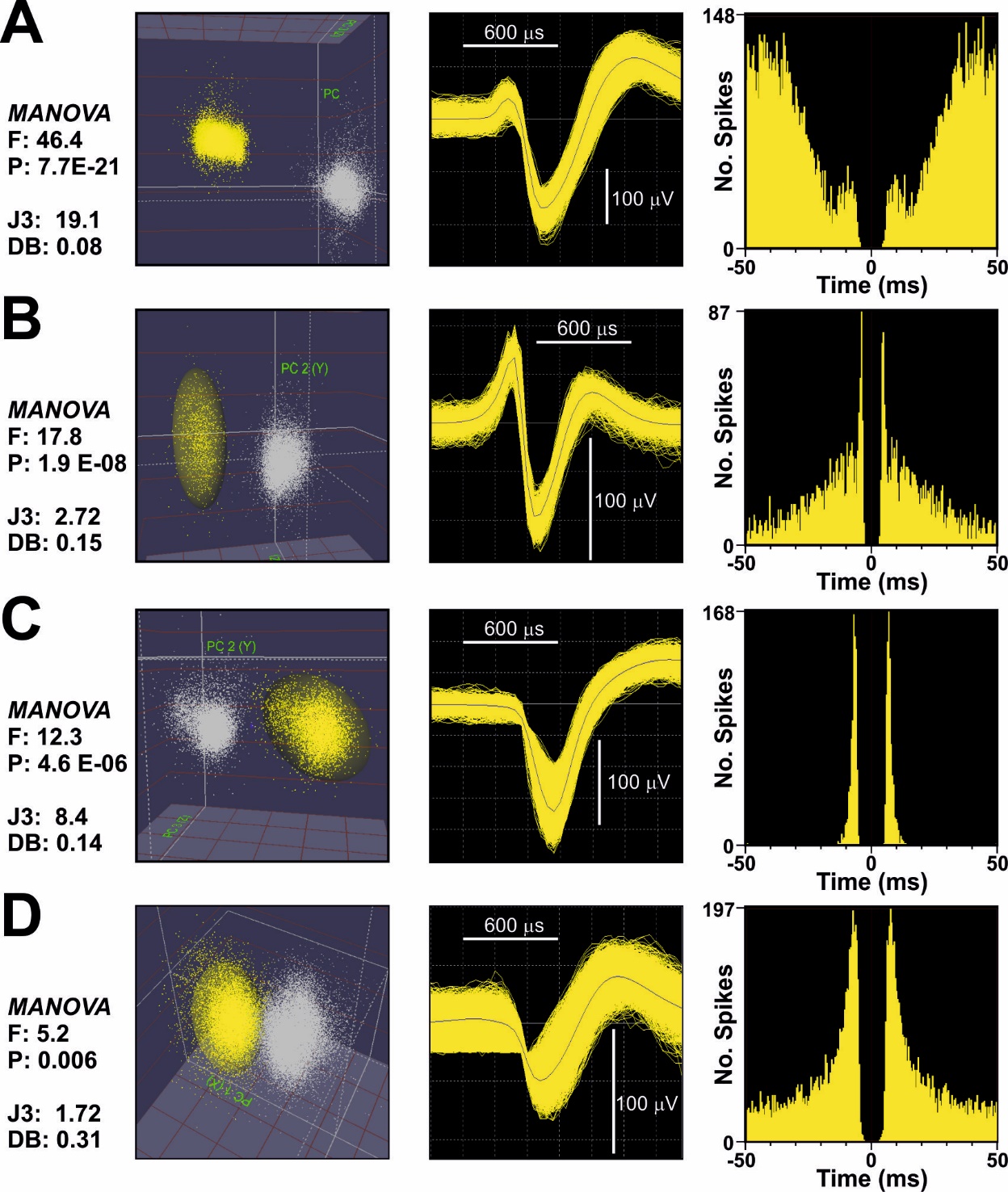


**Supplemental Figure 2. Colour opponent single unit isolation.** (**A-D**) Details of single unit isolation for four colour opponent units. Left panels show 3D feature space (principle components 1-3) for isolated unit (yellow) and unsorted multiunit spikes at that channel (grey), alongside results of MANOVA analysis and J3 and Davies-Bouldin sort quality metrics. Centre panels show overlaid waveforms for all spikes ascribed to the isolated unit. Right panels show unit autocorrelelogram illustrating a clear refractory period. Units in **A** and **B** were classified as S-ON/L-OFF (unit in **A** corresponds to example cell in Fig 1F). Units in **C** and **D** were classified as L-ON/S-OFF (unit in **C** corresponds to example cell in Fig 1G).
